# Incidental Diagnosis of ST-Elevation Myocardial Infarction on Computed Tomography in a Burn Patient: A Case Report

**DOI:** 10.5811/cpcem.53234

**Published:** 2026-07-29

**Authors:** Maria Jose Araujo, Eknoor Sandhu, Angel Romero, Jugraj Randhawa, Rachel O’Donnell

**Affiliations:** * Kern Medical, Department of Emergency Medicine, Bakersfield, California; † University of California, San Diego, School of Medicine, La Jolla, California

**Keywords:** ST-elevation myocardial infarction, computed tomography, radiology, incidental finding, case report

## Abstract

**Introduction:**

ST-elevation myocardial infarctions (STEMI) represent complete occlusion of a coronary artery. An electrocardiogram (ECG) is a method of diagnosis; however, on rare occasions clues of myocardial infarction are first noted on imaging. This is a case of myocardial infarction first noted on computed tomography (CT).

**Case Report:**

A 62-year-old man was brought to the emergency department after being found unresponsive with multiple burns. An ECG performed on arrival did not show changes consistent with myocardial ischemia. Due to reported trauma, a CT incidentally found a heterogeneous low-attenuation area in the posterior wall of the left ventricle of the heart. A repeat ECG subsequently showed an inferior STEMI.

**Conclusion:**

Computed tomography of the chest obtained in the evaluation of trauma may demonstrate evidence of myocardial ischemia. This case highlights the ability of CT to detect cardiac hypoperfusion. The role of CT in myocardial infarction diagnosis remains limited; however, given that the timing of contrast injection depends on the clinical question, it may not reliably assess the hypoperfusion.

## INTRODUCTION

ST-elevation myocardial infarctions (STEMI) are a life-threatening condition. It is the most common cause of morbidity and mortality worldwide and requires immediate intervention.^1^ Patients with atypical symptoms and inconclusive electrocardiogram (ECG) findings can lead to a delay in diagnosis and ultimately poor clinical outcomes.^2^,^3^ Following the detection of a STEMI, prompt coronary angiography with percutaneous coronary intervention is the mainstay of treatment. While computed tomography (CT) is not the typical means of diagnosing myocardial infarctions (MI), it is the imaging modality of choice in the diagnosis of aortic dissection and pulmonary embolism and in the evaluation of trauma.^4^ We present a case of a STEMI that was diagnosed on CT after an initial nonischemic ECG.

## CASE REPORT

A 62-year-old male with no past medical history was transported by ambulance after being found unresponsive. The patient had initially reported to emergency medical services that he had consumed a large amount of alcohol and fallen asleep on the ground. On arrival, his vitals were the following: temporal temperature, 36.6 °C; heart rate, 90 beats per minute; blood pressure, 120/85 mm Hg; respiratory rate, 31 breaths per minute; and oxygen saturation, 94% on room air. He appeared clinically intoxicated. His physical exam was significant for superficial partial burns and deep partial burns over 20–25% of his body, including his back, buttocks, bilateral posterior arms, and bilateral posterior calves. It was suspected that the patient had obtained the burns from lying on hot asphalt for an unknown period, as outside daytime temperatures at this time were consistently above 100 °F.

**Image 1:**
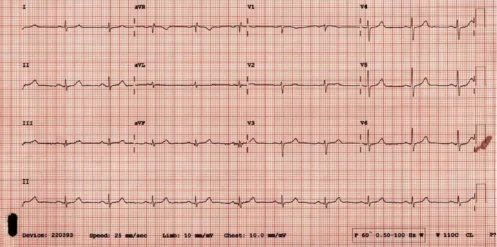
Electrocardiogram performed 2.5 hours after arrival of a 62-year-old man found unresponsive with multiple burns. No ST-segment changes suggestive of acute ischemia were observed.

Treatment was supportive until arrangements were made for transfer to a burn center. Per the Parkland burn formula, the patient was started on 2 L of lactated Ringer’s solution for fluid resuscitation. His burns were covered in saline-soaked gauze, and he received several doses of intravenous (IV) fentanyl for pain control. He was given a tetanus booster and cefazolin for prophylaxis. At that time, the patient’s only complaint was back pain at the location of his burns. Given the ambiguity of the presentation, an ECG was performed two and a half hours after arrival. No ST-segment changes suggestive of acute ischemia were observed on the ECG (Image 1).

**Image 2:**
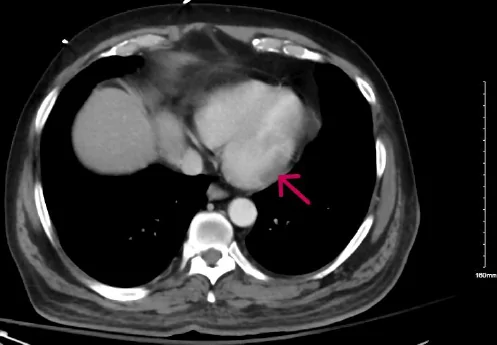
Computed tomography of chest with intravenous contrast showing findings consistent with possible ischemia versus motion artifact to the left heart. The red arrow points to the area of hypoperfusion located in the left lateral ventricle base.

As the patient’s altered mental status resolved, he reported a possible history of trauma from falling out of a truck. He underwent CT with IV contrast of the chest, abdomen, and pelvis five hours after arrival. The radiologist noted a heterogeneous low-attenuation area in the posterior wall of the left ventricle suspicious for ischemia (Image 2).

**Image 3:**
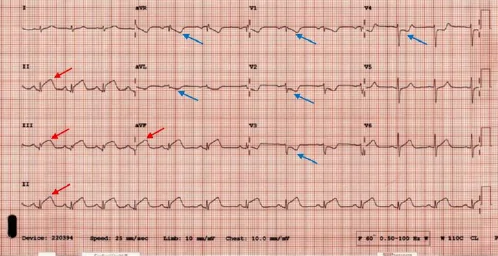
Electrocardiogram indicating a new ST-elevation myocardial infarction. Red arrows point to the new ST elevations, and blue arrows point to the reciprocal depressions.

After returning from CT, the patient did not volunteer any new symptoms. However, the nurse noticed telemetry changes, prompting a repeat ECG 30 minutes after returning from CT, which demonstrated a new inferior STEMI (Image 3).

After the repeat ECG was done, the patient was specifically questioned whether he had developed chest pain, which he confirmed. He was immediately transferred to a STEMI receiving center for percutaneous coronary intervention, where he was diagnosed with an acute inferolateral MI. Coronary angiogram revealed a left circumflex artery occlusion and underwent successful revascularization.

*CPC-EM Capsule*

What do we already know about this clinical entity?

*Myocardial infarction can be incidentally found on computed tomography (CT) chest and CT angiography chest.*

What makes this presentation of disease reportable?

*Myocardial infarction is rarely identified on imaging prior to having telemetry/electrocardiogram changes.*

What is the major learning point?

*All diagnostic workup, whether imaging or labs, can unveil a wide range of incidental findings that must be addressed.*

How might this improve emergency medicine practice?

*This case serves as a reminder to physicians to remain cognizant of unexpected complications during patient care.*

## DISCUSSION

There are a few case reports in the literature of STEMIs incidentally found on imaging. In these cases, patients had either a presentation concerning for cardiac pathology or had multiple comorbidities that increased their risk of an MI. The reports also include CT chest or CT angiography chest to evaluate for cardiovascular or pulmonary vasculature pathology rather than for trauma.^1^ The timing of contrast for trauma is less likely to assess myocardial perfusion abnormalities. Our patient did not endorse cardiovascular risk factors, nor was his initial presentation concerning for MI. There are also known artifacts that may mimic the low perfusion appearance of MI on CT. When the region of decreased perfusion is in the distribution of a coronary artery, then it is more likely to represent infarct rather than artifact.^5^

Computed tomography has been shown to detect acute MIs with a sensitivity rate of 93% and a specificity of 87%, indicating their diagnostic potential.^6^ Furthermore, retrospective case series studies have focused on patients who underwent emergent treatment following incidental cardiac perfusion defects found on CT. The number of myocardial segments on CT with suspected hypoperfusion was inversely correlated to the mean time before starting invasive coronary angiography and emergent cardiological workup, suggesting incidental findings on CT could have a positive impact on intervention timelines.^7^ Contrast-enhanced CT should not replace ECGs and troponin as the primary means to diagnose MIs because they are far more resource-intensive and time consuming. Instead, this case highlights the importance of full interpretation of all diagnostic studies obtained, such as the ability to recognize the subtle signs of cardiac hypoperfusion on a trauma CT.

## CONCLUSION

This case is novel in that an MI was first detected on CT in the assessment of blunt trauma. Our patient’s diagnosis might have been delayed without the astute eye of the radiologist, as the patient did not volunteer the complaints of chest pain. However, we recognize that the time-sensitive nature of STEMIs makes it difficult to do further research on the presentation of acute myocardial ischemia on CT, since delaying treatment to obtain a CT is unacceptable. As in other case reports, we hope this paper highlights the possibility of incidental findings in contrast-enhanced CT imaging, which ultimately lead to the treatment of MI. More importantly, it serves as a reminder to emergency physicians the importance of maintaining situational awareness beyond the obvious clinical presentation, especially in patients who are unable to provide a reliable history.
